# Massive Pulmonary Thromboembolism in Patients with COVID-19; Report of Three Cases

**Published:** 2020-05-16

**Authors:** Mehdi Pishgahi, Zahra Ansari Aval, Behzad Hajimoradi, Rama Bozorgmehr, Saeed Safari, Mahmoud Yousefifard

**Affiliations:** 1 Cardiology Department, Shahid Beheshti University of Medical Sciences, Tehran, Iran; 2 Cardiac Surgeon , Modares hospital, Shahid Beheshti University of Medical Sciences, Tehran, Iran; 3 Electrophysiologist, Shohadaye Tajrish Hospital, Shahid Beheshti University of Medical Sciences, Tehran, Iran.; 4 Clinical Research Development Unit, Shohadaye Tajrish Hospital, Shahid Beheshti University of Medical Sciences, Tehran, Iran.; 5 Proteomic Research Center, Shahid Beheshti University of Medical Sciences, Tehran, Iran; 6 Emergency Department, Shohadaye Tajrish Hospital, Shahid Beheshti University of Medical Sciences, Tehran, Iran; 7 Physiology Research Center, Iran University of Medical Sciences, Tehran, Iran

**Keywords:** Pulmonary embolism, COVID-19, severe acute respiratory syndrome coronavirus 2, venous thromboembolism, thrombolytic therapy, clinical deterioration

## Abstract

COVID-19 is a novel infectious disease, which has challenged people all around the world. As of today, healthcare practitioners and researchers have made great effort to understand the characteristics and clinical presentations of the disease; however, the existing literature is still incomplete in this regard. A growing body of evidence indicates that coagulopathies and thromboembolic events are of utmost importance in COVID-19 patients and are related to poor prognosis. Here, we report three ICU admitted cases of COVID-19, in which massive pulmonary thromboembolism (PTE) occurred a few days after disease onset. Unfortunately, one of the patients did not survive and two were treated; one with thrombectomy and other with antithrombotic agents. It seems that severe cases of COVID-19 are at risk for developing PTE and in-charge physicians should be prepared and plan for anticoagulant prophylaxis using low-molecular-weight heparin (LMWH).

## Introduction

Nowadays, people around the world are facing a devastating challenge, the coronavirus disease 2019 (COVID-19). Since the announcement of COVID-19 pandemic in March 2020, the disease has travelled to every continent, except Antarctica, affecting 2,181,308 people as of April 16, 2020 (1). However, despite the high rate of infected people worldwide, inadequate information about the disease characteristics and presentations is a concern for healthcare providers. 

One of the major concerns regarding COVID-19 patients is the occurrence of thromboembolic accidents. Past studies have demonstrated an increased risk of venous thromboembolism (including pulmonary thromboembolism) in patients with an acute infection (2). With hospitalization being another risk factor for the occurrence of venous thromboembolism (3), the concern seems reasonable. Moreover, some studies have reported coagulation dysfunctions in patients infected with SARS-CoV-2 (4-6), raising even more concern. Also, a study performed by Klok et al. reported that the incidence of thrombotic complications in a group of critically ill COVID-19 patients was considerably high (7). These studies indicate that is important for healthcare providers to bear in mind that occurrence of thromboembolic incidents in COVID-19 patients is possible. 

Here, we present three confirmed cases of COVID-19 (with RT-PCR and Chest computed tomography (CT) scan), admitted to the intensive care unit (ICU) of Shohadaye Tajrish Hospital, Tehran, Iran, with sudden onset of symptoms such as acute decrease in blood pressure and dyspnea. Using pulmonary CT angiography, massive embolism was diagnosed as the reason for their complications.

## Case Presentation:

***Case 1***

**Figure 1:**
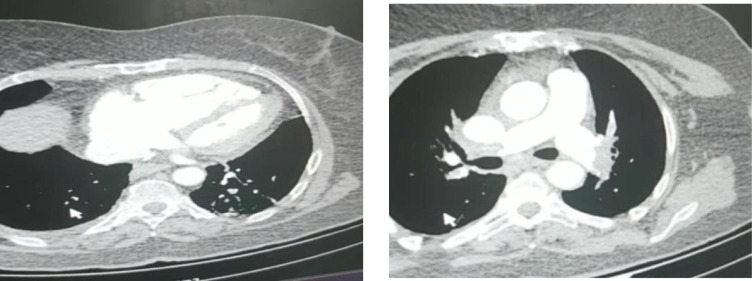
Pulmonary computed tomography angiography of case 1

The first case was a 64-year-old female admitted with an initial symptom of dyspnea (body Mass Index (BMI) = 33 kg/m^2^). The vital signs upon admission were as follows: Blood pressure (BP) = 125/75 mmHg, O_2_ saturation (O_2_Sat) = 82% (in room air), respiratory rate (RR) =24/minute and heart rate (HR) = 125/minute. The patient’s disease history included diabetes and her drug history comprised of Lithium, olanzapine and sodium valproate. She tested positive for COVID-19 and treatment with hydroxy chloroquine, Oseltamivir and Azithromycin was started for her. On day 4 of admission, the patient experienced a sudden drop in BP, with the systolic BP reaching 55 mmHg, and O_2_ Saturation reducing to 70-75%, she also had severe dyspnea. At this time the creatinine level of the patient was 1.8mg/dL. Critical management was promptly initiated and, with suspicion to pulmonary thromboembolism (PTE), pulmonary CT angiography was also performed. The CT angiography revealed severe right ventricular (RV) dilation and massive PTE (Figure 1). Meanwhile, echocardiography showed severe RV dilation, pulmonary artery systolic pressure (PASP) of about 65mmHg and McConnell’s sign. Heparin and Alteplase treatment were immediately started for the patient with a dose of 100mg in two hours. After two hours, the BP was measured, which had reached 110/75 mmHg. The echocardiography performed after the treatment revealed resolution of McConnell’s sign and severe RV dilation, and showed a PASP of about 40mmHg. During the following four days, patient’s overall status improved and her O_2_Sat reached normal levels. The patient was discharged seven days after the anticoagulant treatment, with further oral prescriptions.

***Case 2***

**Figure 2:**
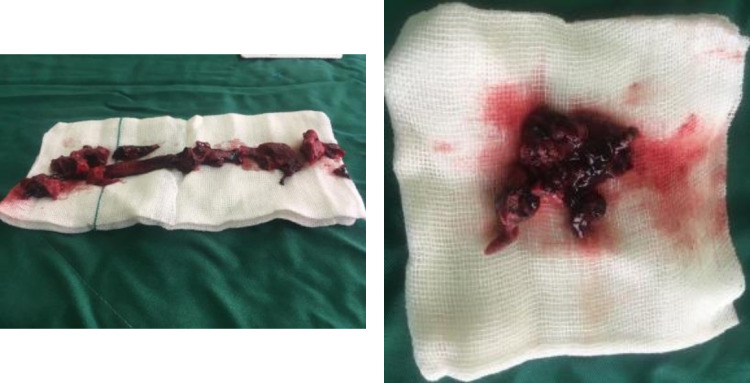
Pulmonary artery clot (left) and right ventricle clot (right) of case 2 extracted via thrombectomy

The second case was a 62-year-old man who also admitted with the initial symptom of dyspnea. Vital signs at the time of admission were as follows: BP = 130/80 mmHg, O_2_Sat = 80%, RR = 22/minute, and HR = 123/minute. Patient’s history evaluation revealed a brain aneurysm surgery, two months prior to the admission. Further evaluations confirmed COVID-19 infection, and treatment with Kaletra and Oseltamivir was started for the patient. On day 4 of admission, suddenly the patient’s status started deteriorating, with his dyspnea worsening and his systolic BP reaching 60-70 mmHg. However, with intravenous (IV) fluid administration, systolic BP increased to 90 mmHg. Bedside echocardiography was performed for the patient, revealing severe RV dilation, RV dysfunction, PASP of 55mmHg, McConnell’s sign and presence of two mobile massive RV clots. Pulmonary CT angiography was also performed, confirming massive pulmonary embolism. As a result, Heparin was administered for the patient, promptly; however, due to the prior history of brain aneurysm, fibrinolytic treatment was not administered, and the patient underwent heart surgery and thrombectomy for removing the clots (figure 2). Later on, the patient’s overall health started to improve, leading to his discharge on day 12 of admission, with prescriptions of anticoagulant agents.

***Case 3***

The last case was a 67-year-old man, hospitalized with symptoms indicative of COVID-19. The patient’s vital signs were as follows: BP = 155/90 mmHg, O_2_Sat = 82%, RR = 25/minute and HR = 130/minute. The patient had a history of hypertension and smoking cigarettes. With confirmed diagnosis of COVID-19, treatment with hydroxy chloroquine, Oseltamivir and Azithromycin was started for the patient. On day 6 of his admission, he began experiencing a decrease in systolic BP, reaching about 60 mmHg, and respiratory distress. The patient was promptly assessed for thromboembolism and CT angiography demonstrated massive pulmonary thromboembolism. However, before any further treatment, the patient went on a cardiac arrest, and after an unsuccessful cardiopulmonary resuscitation (CPR) attempt, he died.

**Figure legends**

## Discussion

The above-mentioned cases may reveal the potential risk of thromboembolic incidents among COVID-19 patients. 

The incidence of massive thromboembolic accidents is a growing concern among healthcare providers regarding COVID-19 patients. Recently, many studies have shown an abnormal portion of the patients, developing life threatening thromboses. However, the underlying cause is still unknown. Some studies have proposed that the cause could be the mechanism of action of the virus. The severe acute respiratory syndrome–coronavirus 2 (SARS-CoV-2) infects host cells via ACE2 receptor (8), which is abundant in different body tissues including the cells lining the blood vessel walls (9). This could mean that the coronavirus is capable of damaging blood vessels, hampering their ability to inhibit clot formation. Some other studies have also drawn attention to prominent and worrisome coagulation changes in COVID-19 patients (10). As a result, clot formation could be one of the worrisome characteristics, associated with COVID-19 infection, and if confirmed, health practitioners should closely monitor and observe for symptoms indicating development of blood clots in COVID-19 patients.

## Conclusion:

Based on the findings, it seems that thromboembolic events should be considered as a potential cause of clinical deterioration in COVID-19 cases and in-charge physicians should consider PTE as a differential diagnosis for worsening of dyspnea in these cases**.**
